# Self-powered triboelectric nanogenerator sensor for detecting humidity level and monitoring ethanol variation in a simulated exhalation environment

**DOI:** 10.1038/s41598-024-51862-6

**Published:** 2024-01-18

**Authors:** Nima Mohamadbeigi, Leyla Shooshtari, Somayeh Fardindoost, Mohaddese Vafaiee, Azam Iraji zad, Raheleh Mohammadpour

**Affiliations:** 1 Center for Nanoscience and Nanotechnology, Institute for Convergence Science and Technology, Sharif University of Technology, Tehran, Iran; 2 Faculty of Engineering, Department of Mechanical Engineering, University of Victoria, P.O. Box 1700 STN CSC, Victoria, BC V8W 2Y2 Canada; 3 Department of Physics, Sharif University of Technology, Azadi Street, P.O. Box 11365-9161, Tehran, Iran

**Keywords:** Energy harvesting, Devices for energy harvesting

## Abstract

Respiration stands as a vital process reflecting physiological and pathological human health status. Exhaled breath analysis offers a facile, non-invasive, swift, and cost-effective approach for diagnosing and monitoring diseases by detecting concentration changes of specific biomarkers. In this study, we employed Polyethylene oxide/copper (I) oxide composite nanofibers (PCNFs), synthesized via the electrospinning method as the sensing material to measure ethanol levels (1–200 ppm) in an exhaled breath simulator environment. The integrated contact-separation triboelectric nanogenerator was utilized to power the self-powered PCNFs exhaled breath sensor. The PCNFs-based gas sensor demonstrates promising results with values of 0.9 and 3.2 for detecting 5 ppm and 200 ppm ethanol, respectively, in the presence of interfering gas at 90% relative humidity (RH). Notably, the sensor displayed remarkable ethanol selectivity, with ratios of 10:1 to methanol and 25:1 to acetone. Response and recovery times for 200 ppm ethanol at 90 RH% were rapid, at 2.7 s and 5.8 s, respectively. The PCNFs-based exhaled breath sensor demonstrated consistent and stable performance in practical conditions, showcasing its potential for integration into wearable devices. This self-powered breath sensor enabling continuous monitoring of lung cancer symptoms and facilitating compliance checks with legal alcohol consumption limits.

## Introduction

Respiration stands as a pivotal biomechanical process facilitating gas exchange between organisms and the atmosphere, involving crucial gases and volatile organic compounds^1–3^. Exhaled breath carries physiological insights, enabling non-invasive disease diagnosis and continuous monitoring^2–4^. This analysis encompasses studying breathing patterns and detecting specific biomarkers, assessing parameters like respiration rate and volume^1,2,4–6^. Variations in these indicators serve as vital health markers, aiding in the early detection of conditions like sleep apnea, asthma, and lung cancer^5,7–12^. Exhaled breath carries metabolic byproducts that reach the lungs through the bloodstream, containing over 3500 volatile organic compounds (VOCs) and specific biomarkers^11,13–16^. Detecting changes in these biomarkers aids in diagnosing diseases^1,3,4,6^. For instance, elevated ammonia levels (1–5.4 ppm) indicate kidney syndromes, excess acetone (1.25–25 ppm) suggests diabetes, and high ethanol levels (12.8–1520 ppb) may indicate lung cancer^3,6,7,9–11,13,14,17–19^. Concentrations above 30 ppb of nitrogen monoxide and carbon monoxide signify asthma, while methane signals liver cirrhosis^3,6,7,9–11,13,14,17–19^. With the rise of COVID-19, respiratory analysis has gained importance, contributing to early disease detection, reduced treatment costs, and mortality rates^3,20,21^. This non-invasive, rapid, and cost-effective approach holds significant potential for at-home illness detection and daily physiological monitoring, revolutionizing healthcare accessibility and effectiveness^13,16,22^.

Precision in measuring exhaled breath ethanol is imperative for non-invasive disease diagnosis, public safety, and law enforcement^3,6,7,9–11,13,14,17–19^. Its accuracy facilitates early detection of ailments like lung cancer, enabling timely medical attention^3,6,7,9–11,13,14,17–19^. Ethanol detection acts as a reliable gauge for monitoring alcohol consumption, crucial for cases related to driving under the influence and ensuring road safety^10,15,23^. It maintains workplace safety in hazardous environments, emphasizing the importance of sobriety^10,15,23^. In aviation safety, ethanol level measurement enhances security, verifying pilots and crew suitability for aircraft operation^10,15,23^. This exact measurement substantially aids health monitoring, ensures legal compliance, and prevents accidents across diverse sectors^10,15,23^.

In recent decades, various techniques such as gas chromatography-mass spectrometry (GC/MS), selected ion-flow tube-mass spectrometry (SIFT-MS), thin film transistor (TFT), and others have been employed to analyze exhaled air in respiratory studies^3,6,24,25^. However, these methods are impractical for daily health monitoring due to the need for sophisticated equipment, trained operators, bulky size, high costs, and lengthy diagnosis times^3,6,24,25^. Conversely, for widespread breath analysis in the general population, highly portable and user-friendly detectors are essential^6,16^. Chemo-resistive gas sensors based on semiconducting metal oxides have emerged as a promising technique for breath analysis^3,8,16,19,26–33^. They offer simplicity, affordability, ease of manufacture, rapid response, low power consumption, and miniaturization potential for portable devices^3,8,16,19,26–33^. Many existing breath analysis methods rely on external power sources, with conventional batteries being the predominant choice^6,16^. However, batteries suffer from issues such as bulkiness, limited lifespan, high costs, and chemical contamination, hindering the development of portable and sustainable real-time respiration monitoring devices^3,15,34^. To address these challenges, a compelling solution involves the creation of self-powered devices that can harness energy from various sources, including human biomechanical energy, solar power, thermal energy, and others^3,15,34^.

In contrast to other energy harvesting methods like solar cells, piezoelectric, and thermoelectric systems, triboelectric nanogenerators (TENGs) offer distinct advantages, being lightweight, high energy density, cost-effective, sensitive in detecting changes, and biocompatibility, allowing various materials to be used for self-powered breath monitoring sensors^2,3,21,35–39^. TENG is a promising energy harvesting technology and a renewable energy source developed by Zhong Lin Wang’s group in 2012^40^. It operates through triboelectrification and electrostatic induction, converting mechanical energy from the environment and human biomechanical movements into electrical energy^37,41^. Integrating TENGs with sensors has led to the creation of advanced self-powered devices, including respiratory analysis sensors, pulse wave detectors, pressure, and velocity sensors^1–3,5,14,34,36,38,39,42–53^. One notable achievement is the development of a self-powered breath sensor, where gas sensors are combined with TENGs to detect chemical compounds and biomarkers in exhaled gases^3,5,34^. This non-invasive diagnostic approach finds applications in monitoring physiological parameters and indicating diseases like diabetes, cancer, and COVID-19^2,14,21,36,38^. In the following, a number of studies endeavors pertaining to respiratory sensors in diverse fields of application have been reviewed. Wang et al. devised a respiration-driven TENG based on Ce-doped ZnO-PANI nanocomposite film for the detection of multiple respiratory parameters including trace-level NH_3_ concentration, human respiratory flow, and respiratory frequency^14^. Shan et al. developed a self-powered humidity sensor to monitor human breath states using a flexible TENG based on Ti_3_C_2_T_x_: PDMS composite film and Ni/Cu polyurethane foam sponge, and RGO-TiO_2_ as a humidity sensor^1^. In another study, Lu et al. designed an intelligent facemask based on TENG to monitor individuals' breathing patterns and served as a diagnostic tool for respiratory illnesses, including the COVID-19 pandemic^21^. Zhang et al. developed a breath-driven TENG for self-powered human–machine interaction (HMI). The TENG uses a PET film to harvest energy from breathing and functions as a self-powered sensor in a smart wireless breath-driven HMI system with signal processing^54^. Su et al. designed a self-powered alveolus-inspired membrane sensor by utilizing a WO_3_/copper-coated acrylic sheet. This sensor demonstrates a dual functionality by enabling continuous monitoring of breath behavior and detecting NO_2_ at room temperature^5^.

In this study, we explored variations in sensing parameters within an environment mimicking human exhaled breath associated with lung cancer, encompassing varying ethanol concentrations at 90 RH%. Ethanol concentration alterations in exhaled breath are indicative of early-stage lung cancer and are also a reliable marker for detecting cases of driving under the influence^10,15,23^. To achieve this, nanofiber structures synthesized using the electrospinning method were employed as exhaled breath sensors on gold-sputtered interdigitated electrodes featuring a 100 μm gap and a 2 cm × 2 cm area. Two types of nanofiber structures, namely polyethylene oxide nanofibers (PNFs) and polyethylene oxide/copper (I) oxide composite nanofibers (PCNFs), served as active materials in the breath sensor, tested under varying conditions of humidity, ethanol in dry air, and 90 RH% (simulating exhalation)^1,10^. Polyethylene oxide, known for its hydrophilic nature and porous structure, facilitates water and ethanol molecule adsorption, making it ideal for sensor applications. Despite their advantages, polymeric materials often lack sensitivity and stability, making them unsuitable for breath analysis. However, copper (I) oxide nanoparticles exhibit excellent gas sensing properties due to their extensive surface area and abundant adsorption sites in its cationic part, resulting in superior performance. Integrating these materials into a nanofibrous network enhances gas molecule adsorption sites, boosting charge carrier production through increased water and ethanol absorption, thus amplifying electrical conductivity changes. The sensor exhibited a rapid response rate of 3.2 to a 200 ppm ethanol concentration in the presence of 90 RH%, even amidst potent interfering gases. Evaluation criteria included selectivity in methanol and acetone atmospheres, long-term stability at 200 ppm and 5 ppm ethanol concentrations within 90 RH%, output signal repeatability at 200 ppm ethanol and 90 RH%, as well as response/recovery times in this specific environment. Additionally, mechanisms influencing sensing performance in diverse settings were thoroughly investigated. To make a self-powered sensor, the FTO/Kapton triboelectric nanogenerator was utilized as the power source, and its output signal changes were studied in different atmospheres. Ultimately, the PCNFs' sensing performance was rigorously evaluated in the practical context of human breath analysis. The sensor developed in this study possesses key attributes, including scalability, lightweight design, flexibility, biocompatibility, and suitability for wearables. Furthermore, integration with a triboelectric source has enabled the creation of a portable and wearable self-powered sensor.

## Experimental section

### Fabrication and characterization of exhaled breath sensor

**Figure 1:**
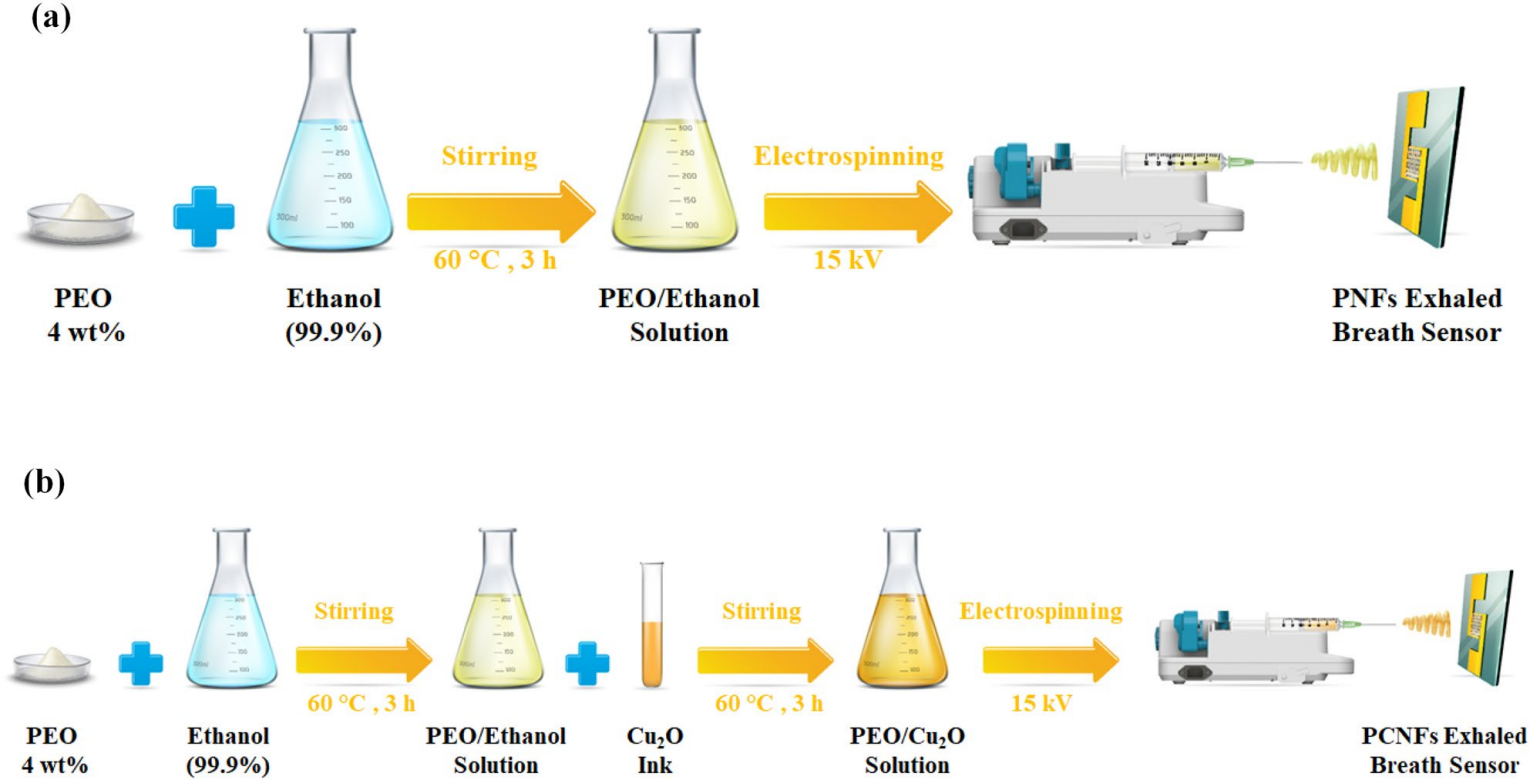
Synthesis procedures of exhaled breath sensors: (**a**) Polyethylene Oxide Nanofiber (PNFs) Sensor, and (**b**) Polyethylene Oxide/Copper (I) Oxide Composite Nanofibers (PCNFs) Sensor.

Exhaled breath sensor was fabricated using the electrospinning method to produce polyethylene oxide nanofibers (PNFs) and polyethylene oxide/copper (I) oxide composite nanofibers (PCNFs) from their solutions as the sensing materials. Initially, ethanol (99.9% ≤ , Sigma Aldrich) was heated to 60 °C, and then 4 wt% of polyethylene oxide (M_w_ = 1,000,000 g/mol, powder, Sigma Aldrich) was added and stirred for 3 h to obtain a homogeneous solution for PNFs production. The utilization of high molecular weight polyethylene oxide results in reduced solubility in water at room temperature, necessitating prolonged stirring for complete dissolution. This characteristic enhances the sensor's stability against degradation in high humidity conditions. Similarly, a 4 wt% solution of polyethylene oxide/ethanol was prepared and then copper (I) oxide ink (nanospheres dispersion in ethanol, < 300 nm particle size, Sigma Aldrich) was added at 60 °C and stirred for 3 h to prepare a homogeneous solution for the synthesis of PCNFs. The prepared solutions were then separately poured into 5 ml syringes with 19 gauge and converted into desired nanofibers using an electrospinning device (Lab2 ESI-II, Nanoazma Inc., Iran) under the conditions of 15 kV spinning voltage, 10 cm tip-collector distance, and 1 ml/h flow rate, maintained for 20min. Gold-sputtered interdigitated electrodes with a gap of 100 μm and 2 cm × 2 cm area on a glass slide, were employed as a substrate to collect PNFs (Fig. 1a) and PCNFs (Fig. 1b). Finally, the electrospun nanofibers were dried in an oven at 60°C for 5h to remove ethanol and obtain the required sensors.

Field emission scanning electron microscope (FESEM) and energy dispersive spectroscopy (EDS) were employed to characterize the morphology of the PNFs and PCNFs using Tescan (MIRA3, 5 kV). The crystal structure of the PCNFs was investigated using X-ray diffraction (XRD) pattern analysis with a PAN analytical X’Pert PRO instrument in the range of 20–70 degrees.

### TENG structure

The integrated contact separation triboelectric nanogenerator (CS-TENG) utilized to power the breath sensor consisted of FTO glass and Kapton with an aluminum tape behind it, serving as the back contact layer. A homemade machine was designed to investigate the performance of FTO/Kapton-based TENG in mechanical harvesting energy, as depicted in Fig. S1. A linear electric motor capable of controlling the applied force via the stepping position was developed and used during the electrical measurement. The behavior of the CS-TENG was investigated by adjusting the motion of the two electrodes using gauges on a vertical contact-separation tapping device at different frequencies ranging from 1 to 4 Hz, and applying a force of 6 N to bring the two surfaces into contact, which leads to the induction of triboelectric charge. The maximum spacing distance of the electrodes was 2 cm, and the effective contact area of the TENG was 8 cm × 8 cm. The voltage and current of the TENG-based system based were measured using a DSO1022A digital oscilloscope (Agilent Technologies) and a potentiostat–galvanostat (µAuto-lab system, Metrohm), respectively.

### Exhaled breath simulator setup and sensing measurements

In this study, the performance of the exhaled breath sensor was rigorously evaluated using a meticulously designed breathing simulator setup under controlled room temperature conditions (25 ± 2 °C). The simulator accurately replicated the varying levels of humidity (ranging from 30 to 99% relative humidity) and ethanol vapor concentrations (ranging from 1 to 200 ppm) at 90 RH%, a condition approximating the humidity levels found in human exhaled breath, thus ensuring the relevance and applicability of the experimental data^1,10^.

**Figure 2:**
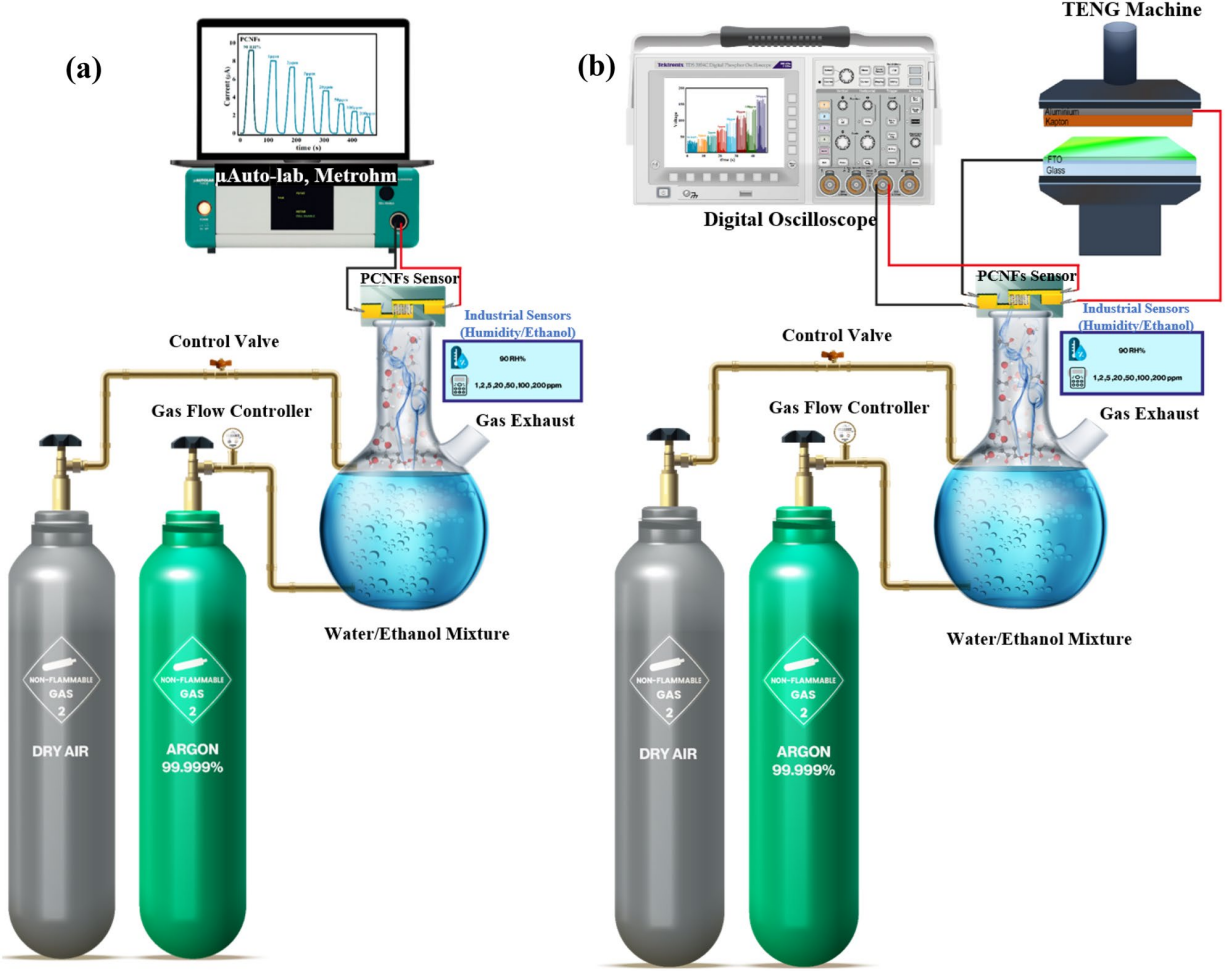
The experimental set-up comprises an exhaled breath simulator intended to evaluate the performance of PCNFs sensors across various environments containing different ethanol concentrations (1, 2, 5, 20, 50, 100, 200 ppm) at 90 RH% through two distinct modes: (**a**) employing an external power source via µAuto-lab system, and (**b**) utilizing a triboelectric nanogenerator for self-powering.

To attain various relative humidity levels within the setup, inlet pure argon gas (99.999%) was precisely directed into deionized water (> 18 MOhm-cm), as depicted in Fig. 2. This process required controlled argon gas flow, utilizing precise regulators and valves to generate environments with predetermined ethanol vapor concentrations and humidity levels. To ensure precision, gas concentrations in the testing environment were continuously monitored using two types of commercial hygrometers and an ethanol sensor strategically positioned near the sensor. Similarly, to regulate different concentrations of ethanol vapor at 90 RH%, argon was blown into a specific volume ratio of ethanol (99.9% ≤ , Sigma Aldrich) and DI-water mixture, calculated based on Henry’s law (The calculation details can be found in the supporting information)^33,55–58^.

For evaluating the sensing parameters of the nanofiber-based exhalation sensor in the atmospheres mentioned above, potentiostat–galvanostat (µAuto-lab system, Metrohm) was connected in series to measure the output I–t variations with a constant bias voltage of 2 V (Fig. 2a). Furthermore, FTO/Kapton TENG was connected in parallel as a source power to construct a self-powered breath sensor, and the output transient voltage changes were recorded by DSO1022A digital oscilloscope in various ambient atmospheres. A bridge rectifier was used to reach a steady voltage from the alternating electric output of the TENG (Fig. S2b).

The sensor response was defined as the ratio of the absolute value of the sensor's electrical resistance variation to the initial sensor resistance (ΔR/R_0_) for different relative humidity and ethanol vapor concentrations. For various ethanol vapor concentrations at 90 RH%, the response was described as ΔR/Rg where ΔR represents R_g_–R_90RH%_ that R_g_ being the sensor resistance at a specific gas concentration and R_90RH%_ indicating the sensor resistance in 90 RH%.Also, sensor's base resistance measures 8 MΩ. The time required for the sensor to reach 90% of the total output signal change upon gas adsorption or desorption was defined as the response time and recovery time, respectively.

## Result and discussion

### TENG characterization

**Figure 3:**
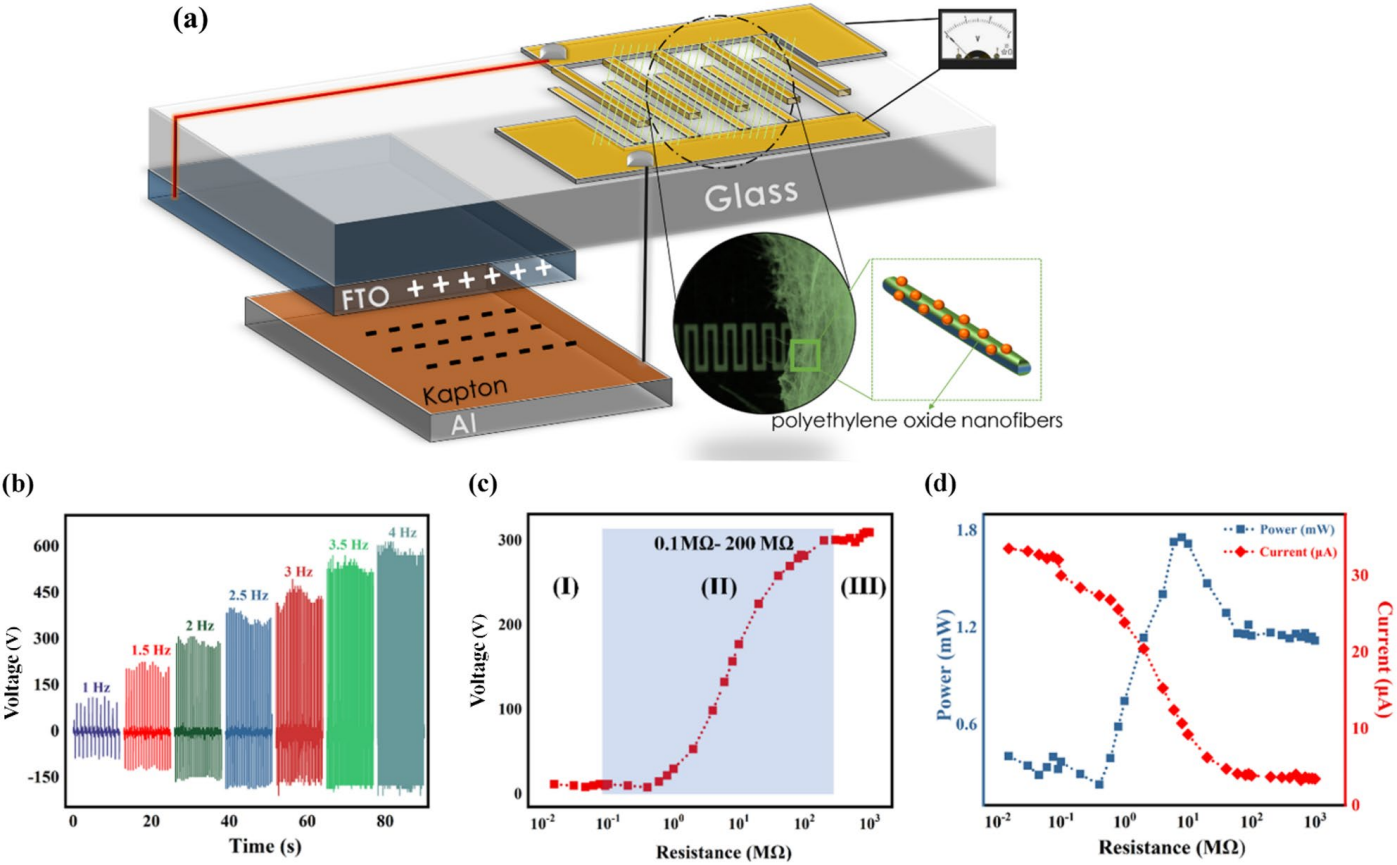
(**a**) Schematics of integrated FTO/Kapton TENG- powered- sensor; (**b**) Open-circuit voltage (V_OC_) of the FTO/Kapton TENG operating under various working frequencies ranging from 1 to 4 Hz; (**c**) The voltage amplitude diversity of the FTO/Kapton TENG vs external load resistance; (**d**) The evolution of power and current of FTO/Kapton TENG versus external load resistance.

The Schematics of the integrated TENG- powered- sensor is illustrated in Fig. 3a.The principle of the electrical current generation of the FTO/Kapton TENG in the contact-separation mode is described in Fig. S2a. Initially, when the FTO and Kapton surfaces come into contact with each other (a-I), opposite triboelectric charges are generated on their surfaces. Upon separation of the electrodes (a-II), a potential drop arises, leading to the induction of charges. This results in positive charges on the aluminum back-contact, which generates electron movement from aluminum to FTO. In the released state, no electric current passes through the circuit, and the system is in equilibrium (a-III). Subsequently, during the pressing process, the reverse displacement of electrons occurred (a-IV). This movement of the induced charges in opposite directions produces two signal peaks with contrary signs and leads to the production of electricity by a tapping-mode TENG as a source power^37,42,46,47^. The open-circuit voltage (V_OC_) results of the FTO/Kapton TENG operating under various working frequencies in Fig. 3b ranging from 1 to 4 Hz and subjected to a uniformly applied force of 50 N is presented in Fig. 3b. The peak value of the V_OC_ exhibits an upward trend with increasing in the tapping frequency and enhances from 138.75 to 592.85 V.

Additionally, the FTO/Kapton TENG output voltage at a frequency of 2 Hz was investigated by varying the load resistance (Fig. 3c). The semi-log plot of output voltage versus load resistance revealed three distinct zones. When TENG provides power to the device through the impedance matching circuit, zone II becomes a critical region^42,44,46^. In this context, the proper coupling of the TENG impedance and the device's resistance occurs when the variation in load resistance falls within the range of the device's resistance^46,49^. Based on Fig. 3c, it is evident that the active zone of the FTO/ TENG is between 0.1 and 200 MΩ when operating at a frequency of 2 Hz. Accordingly, it is anticipated that the proposed self-powered humidity sensor, coupled with the FTO/Kapton TENG, will operate under various frequencies without significant changes in the sensor's output results.

The output power and current of the FTO/Kapton TENG were assessed by applying various external load resistances at a frequency of 2 Hz. As illustrated in Fig. 3d, the peak output current decreased as the resistance increased from 0.1 to 200 MΩ. Additionally, the output power initially enhanced with the increase of the load resistance, reaching a maximum instantaneous power of 1.8 mW at the resistance of ≈ 10 MΩ, and then decreased (Fig. 3d). The output power P was calculated using the formula P = V^2^/R, where V and R represent the output peak voltage in the external load and the load resistance of the TENG, respectively^37^.

### Sensor characterization

**Figure 4:**
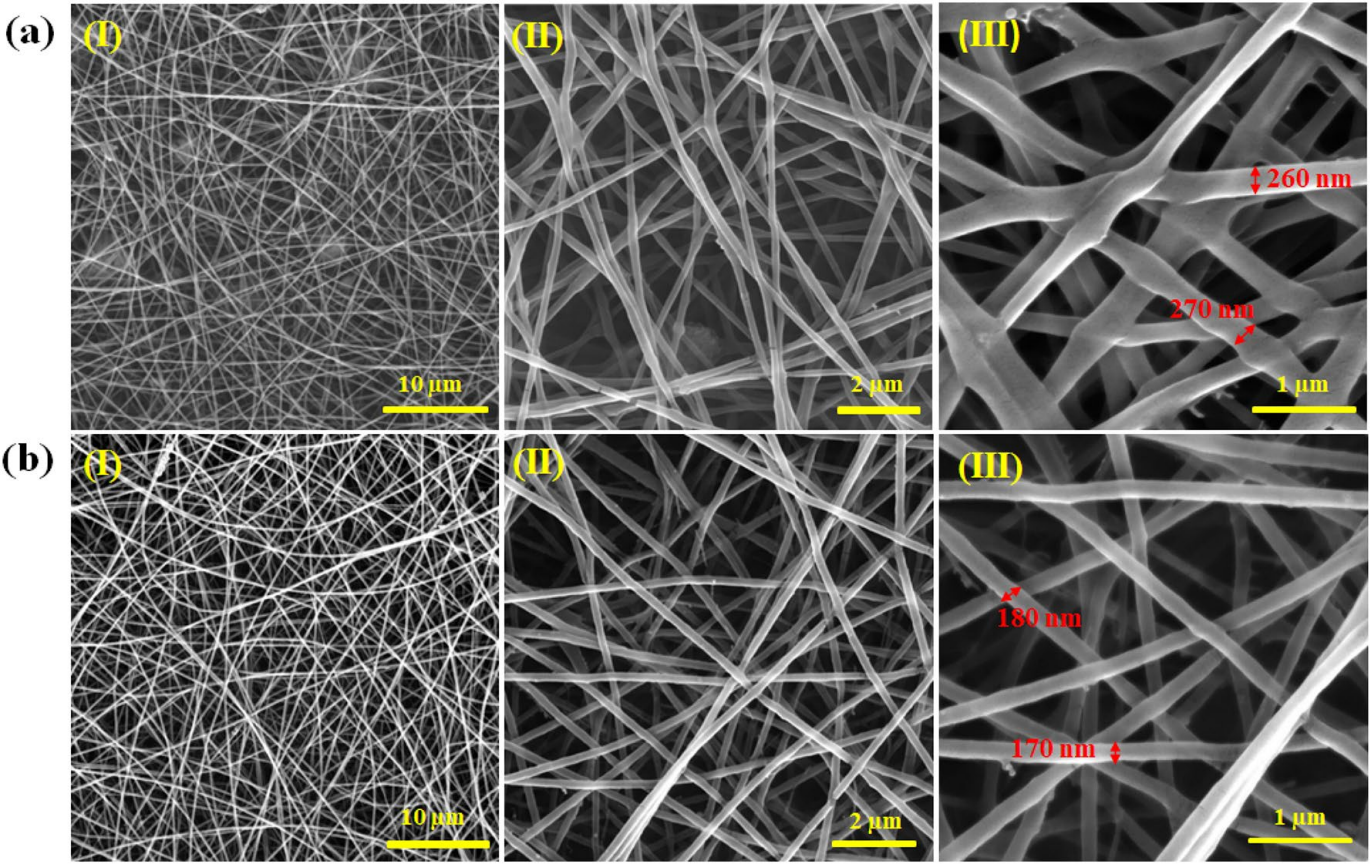
Top view FESEM images of (**a**) polyethylene oxide/copper (I) oxide composite nanofibers (PCNFs); and (**b**) polyethylene oxide nanofibers (PNFs) at different magnifications (**I**) 5 kx, (**II**) 10 kx, (**III**) 50 kx.

The surface morphology of electrospun nanofibers was examined using FESEM. The images obtained from polyethylene oxide/copper (I) oxide composite nanofibers (PCNFs) and polyethylene oxide nanofibers (PNFs) at different magnifications of the FESEM are shown in Fig. 4a,b, respectively. The images reveal that the average diameter of PCNFs (~ 266 nm) is larger than that of PNFs (~ 161 nm) due to the incorporation of copper (I) oxide nanoparticles with a particle size of < 300 nm. Also, the continuity and uniformity of the as-spun nanofibers result in a structure with a high aspect ratio and a large specific surface area, which is essential for achieving a sensitive breath sensor with great responsivity and short response/recovery time.

**Figure 5:**
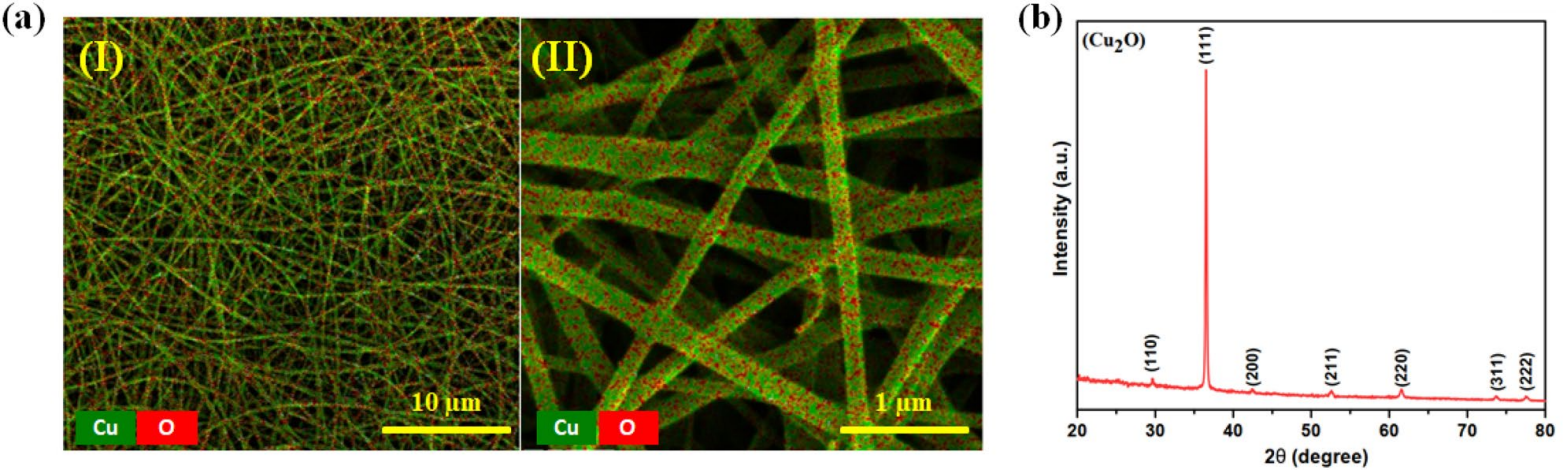
(**a**) EDS of PCNFs at different magnifications (**I**) 5 kx, (**II**) 50 kx. (**b**) XRD pattern of PCNFs.

The FESEM and EDS elemental mapping of PCNFs was shown in Fig. S3 and Fig. 5a at two magnifications. In Fig. 5a, the homogenous dispersion of copper (I) oxide nanoparticles along the nanofiber without other impurity elements is evident from the EDS elemental mapping (green dots represent copper, and red dots indicate oxygen). Moreover, XRD analysis (shown in Fig. 5b) was conducted to investigate the crystal phase of PCNFs. All the characteristic diffraction peaks in the pattern could be indexed well to the copper (I) oxide structure and matched with the standard card (JCPDS No. 05-0667), which suggests a high purity of the product.

### Sensing performance and mechanism

The main goal of this research is to develop a self-powered breath sensor capable of detecting the concentration of ethanol vapor in exhaled breath in two ranges: below 10 ppm for detecting diseases such as lung cancer, and above 10 ppm to serve as critical evidence in discriminating drunk driving cases^38,59^. To achieve this, three atmospheric conditions including humidity, pure ethanol vapor in dry air, and different concentrations of ethanol vapor at 90 RH% (emulating exhaled breath) were employed to evaluate the sensor's performance^1,10^. The sensing signal was assessed by analyzing the alterations in the resistance of the PCNFs and PNFs sensors when subjected to the environments as mentioned above. To this end, two power sources were utilized, namely a potentiostat with a constant bias voltage of 2 V and a triboelectric nanogenerator. To achieve a reliable and portable self-powered exhaled breath sensor, several features such as high sensitivity, low operating temperature, fast response and recovery time, great selectivity, lightweight, and flexibility need to be considered. Composite nanofibers of polymer/metal oxide semiconductors are one of the most promising materials for high-performance sensors^60,61^. In this regard, high surface area copper (I) oxide nanoparticle/polyethylene oxide composite nanofibers and also polyethylene oxide nanofibers were synthesized as sensor materials via the electrospinning method. Adsorption of water molecules occurs on the surface of polyethylene oxide (PEO) nanofiber due to its hydrophilic nature through the formation of hydrogen bonds, resulting in a higher level of electrical conductivity^25,60,62–64^. Polymeric materials, despite their advantages, have certain drawbacks such as poor sensitivity and fluctuations in sensing signals, which are not satisfactory for use in breath analysis and require approaches to overcome these limitations^26,62,63^. Additionally, cuprous oxide (Cu_2_O) is p-type semiconductor catalytic material with an energy band gap of approximately 2.1 eV. It exhibits remarkable gas sensing properties attributed to its extensive specific surface area and abundant active sites in the cationic part for gas adsorption. Notably, it operates at low temperatures, possesses excellent electrical characteristics, is non-toxic, and remains stable in the air, rendering it highly suitable for sensor applications. Water vapor molecules chemisorb onto the active sites, leading to dissociation near these sites. This phenomenon modulates electrical conductivity, triggered by the adsorption and desorption of various gases^27,28,30,65–67^. Moreover, the sensing performance of metal oxide semiconductor-based sensors is greatly affected by the microstructure and morphology of the sensing materials^26,63^. Hence, the design and synthesis of a composite nanofiber network of the hydrophilic polymer and semiconducting oxide with a high specific surface area can provide numerous adsorption sites for gas molecules, leading to the generation of more charge carriers for electrical conduction^24,63,68^. This can significantly enhance the gas sensor efficiency through a synergistic effect^24,63,68^. To this end, we employed PEO/Cu_2_O composite nanofibers (PCNFs), as well as PEO nanofibers (PNFs), synthesized via electrospinning, as active sensor materials in this study.

**Figure 6:**
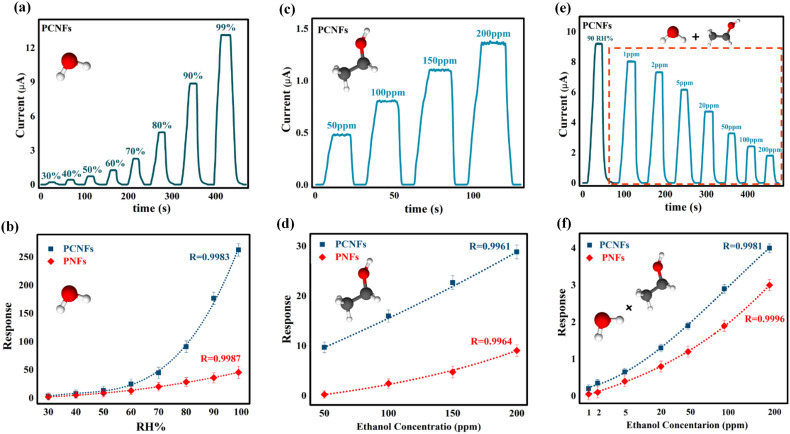
(**a**) Variation in PCNFs current with relative humidity ranging from 30 to 99%; (**b**) Response values of PCNFs and PNFs at relative humidity ranging from 30 to 99%; (**c**) Variation in PCNFs current with ethanol concentrations of 50, 100, 150 and 200 ppm; (**d**) Response values of PCNFs and PNFs at ethanol concentrations of 50, 100, 150 and 200 ppm; (**e**) Variation in PCNFs current with ethanol concentrations of 1, 2, 5, 20, 50, 100, and 200 ppm at 90% RH; (**f**) Response values of PCNFs and PNFs with ethanol concentrations of 1, 2, 5, 20, 50, 100, and 200 ppm at 90% RH.

**Figure 7:**
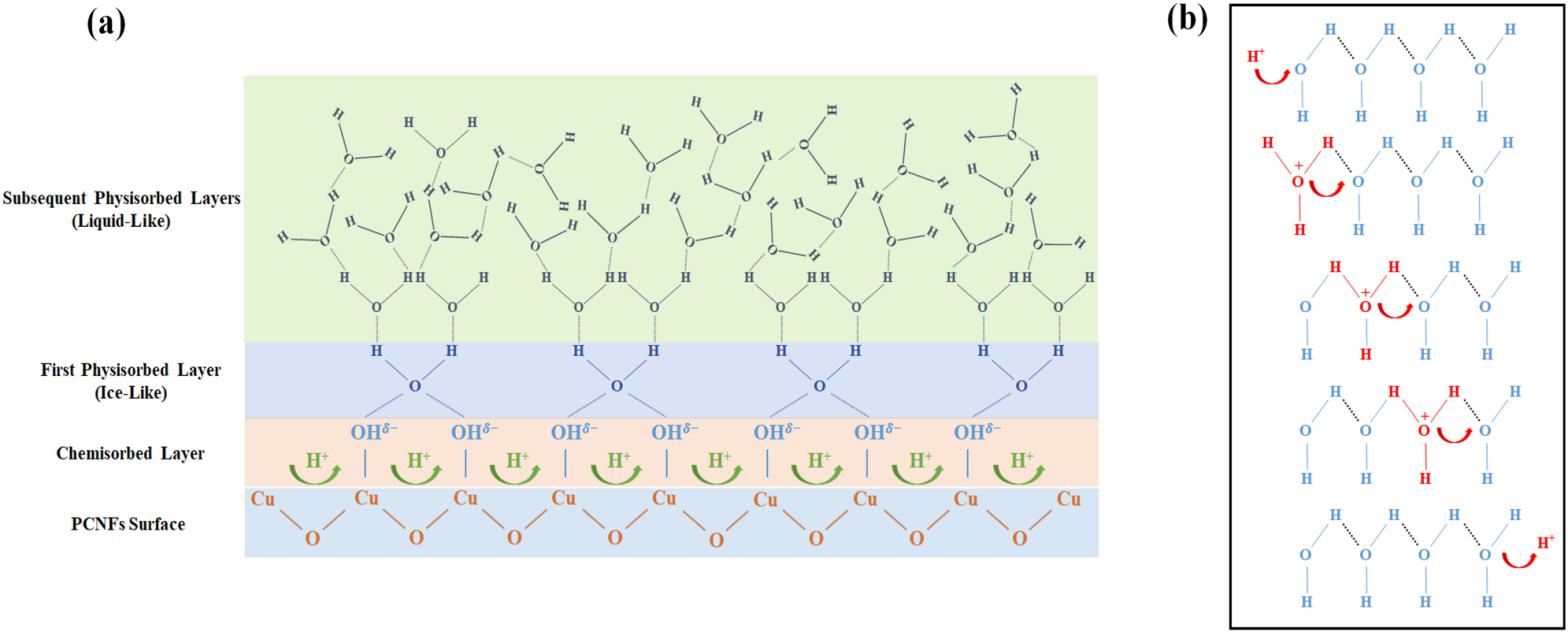
(**a**) Schematic illustration of humidity adsorption process on the PCNFs sensor, and (**b**) Proton hopping mechanism for H^+^ transfer through surface-adsorbed humidity.

The room temperature sensing behavior of the PCNFs and PNFs sensors in the range of 30% to 99% relative humidity was illustrated in Fig. 6a and Fig. S4a, respectively. As depicted, the electric current value for PCNF increased from 0.4 ± 0.05 to 13 ± 0.3 µA by raising the RH% value from 30 to 99%, while it enhanced less for PNFs and reached from 0.1 ± 0.02 to 1.9 ± 0.1 µA with high fluctuation in electrical current signals. In addition, Fig. 6b demonstrates that PCNFs exhibit superior response compared to PNFs to different humidity concentrations, and the response level increases significantly from 60 RH% onwards. In the following, various mechanisms which have been proposed to explain the reason for this type of sensing behavior through PCNFs and PNFs structures are considered^60,62,63,69,70^. Initially, water vapor molecules are chemisorbed onto active sites of the sensing material (i.e., the semiconductor cationic part and the hydrophilic PEO surface) and dissociate near these sites. This leads to the formation of a dipole (Cu^δ+^  − *OH*^δ−^) and mobile proton (H^+^) as a charge carrier (Fig. 7a)^26,60,62,69,70^. By applying the electrical field, the dissociated mobile proton migrates through the hopping between adjacent dipoles and produces the secondary hydroxyl group^26,60,62,69,70^. This results in the surface being covered with hydroxyl ions in the chemisorbed layer at low humidity levels, which forms the foundation for further physisorption of water vapor molecules at higher relative humidity^60,62,63^. After occupying all absorption sites of the sensor surface by hydroxyl ions and thus completing the chemisorption layer, each subsequent water vapor molecule undergoes physisorption onto the two neighboring hydroxyl ions through hydrogen bonding and forms the first physisorption layer^26,60,62,63,69,71^. Water molecules in the first physisorbed layer are constrained by the two hydrogen bonds and cannot move freely or orient themselves under an external electric field^62,63,69,70^. Due to the strong electrostatic fields created by the chemisorbed layer, few physisorbed water molecules dissociate, producing hydrogen ions (H^+^)^62,63,69–71^. The H^+^ ions bind to existing water molecules and produce hydronium ions (H_3_O^+^), which are the dominant ions in the physisorbed layer^60,71^. Subsequently, H_3_O^+^ releases a proton to the adjacent H_2_O molecule and forms another hydronium ion via proton hopping (Fig. 7b)^60,62,63,69,71^. This mechanism is known as the Grotthuss chain reaction and is the primary process that enhances the conductivity of sensing materials at low humidity levels (primary chemisorbed/physisorbed layers)^63,69,71^. The increase in water vapor condensation on the surface results in the formation of an additional layer on the first physisorbed layer, changing it from a single layer to multiple layers^62,63,69^. This layer is less organized than the first physisorbed layer as it may contain only one hydrogen bond that exists locally^62,63,69^. Consequently, the order of the primary layers gradually disappears with the adsorption of water molecules in the subsequent physisorbed layers, forming a liquid-like network that provides more independence for proton transport through the Grotthuss mechanism in these layers (Fig. 7a)^62,63,69,71^. The activation energy required for proton hopping from the second physisorbed layer onwards is reduced, and the Grotthuss mechanism prevails, leading to a rapid increase in conductivity at high humidity levels^43,47,63,69^. At very high humidity levels, in addition to the drastic proton conductivity in the adsorbed layers, the dominant mechanism will be the diffusion of hydronium ions through an electrolyte in the condensed water liquid layer within the micropores^63,69,72^. If the transport is dominated by the diffusion mechanism of hydronium ions, the electromotive force will be three times higher compared to transport dominated by the Grotthuss mechanism^69^. Therefore, according to the mechanisms mentioned above, the conductivity and, subsequently, the response value of the PCNF sensor increases strongly with the increase in relative humidity from 60%^43,47,63,64,69,71,72^. Furthermore, PCNFs possess more active sites for absorbing humidity compared to PNFs due to the simultaneous presence of Cu_2_O and PEO in their structure, which makes their sensing performance much superior and reliable^25,60,62–64^.

The variation of output current signals for PCNFs and PNFs at different ethanol concentrations are presented in Fig. 6c and Fig. S4b, respectively. Consistent with the humidity sensing results, the output current changes for PCNFs are more significant than those for PNFs. As the concentration of ethanol increases from 50 to 200 ppm, the output current of PCNFs rises from 0.5 ± 0.03 to 1.4 ± 0.07 µA, while for PNFs it increases from 0.08 ± 0.01 to 0.48 ± 0.05 µA. Comparing Fig. 6b and d, it is evident that the response level of the PCNFs sensor to variations in ethanol concentration is far lower than its response level to changes in humidity. This can be attributed to the weaker performance of ethanol compared to humidity in the proton hopping process in the Grotthuss mechanism^71,73,74^. Compared to the water molecule structure, the ethanol molecule has a methyl group (-CH_3_CH_2_) instead of a hydrogen atom attached to the -OH group, which results in a larger molecule size and less mobility freedom under the applied electric field^71,73,74^. This factor and the lower polarity of ethanol, lead to weaker proton hopping compared to water vapor and result in a lower electrical conductivity improvement^71,73^. In addition, the performance of the exhaled breath sensor was investigated in a simulated environment with different concentrations of ethanol vapor present at 90 RH%. The lower performance of ethanol in the proton transfer process than water vapor caused disruptions in the humidity network proton hopping. As a result, the sensor's conductivity decreased further as the ethanol concentration increased. As shown in Fig. 6e and Fig. S4c, the electrical current level decreased from 9.2 ± 0.1 to 2 ± 0.1 µA for PCNF as the concentration of ethanol vapor increased from 0 to 200 ppm at 90 RH%, and for PNFs, it reduced from 2.1 ± 0.1 to 0.4 ± 0.05 µA. The response values for PCNF and PNFs (Fig. 6f) at 200 ppm ethanol in 90 RH% were 3.9 and 2.9, respectively.

Overall, the PCNFs sensor exhibited superior and consistent sensory signal and response levels across all tested atmospheres compared to the PNFs sensor, primarily due to the utilization of both hydrophilic polyethylene oxide nanofibers and copper (I) oxide nanoparticles, resulting in a greater number of absorption sites. This observation highlights the potential of the PCNFs sensor for further investigation and development as a promising exhaled breath sensor for various applications.

**Figure 8:**
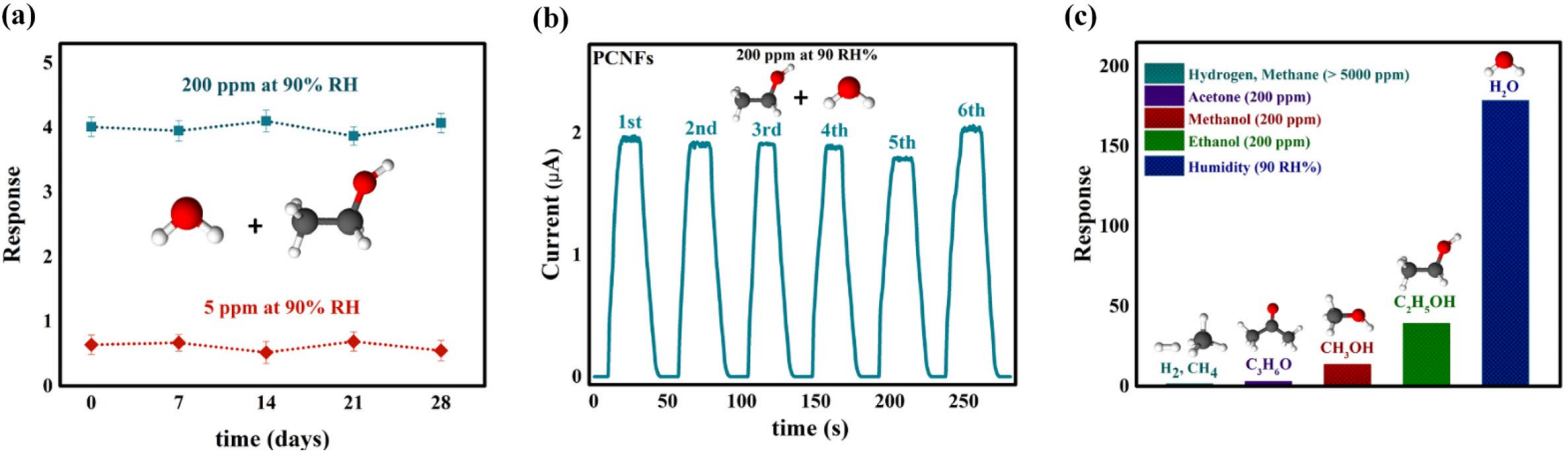
(**a**) Stability of the PCNFs sensor response to 5 ppm and 200 ppm ethanol concentrations at 90 RH% environments from the day of fabrication to every 7 days over 4 week; (**b**) Repeatability of the PCNFs sensor response to 200 ppm ethanol at 90 RH% for 6 repetitions; (**c**) Selectivity of the PCNFs sensor against ethanol, methanol, acetone, hydrogen, methane, carbon monoxide, and moisture.

It is well known that long-term stability for gas sensors that operate in high humidity environments, such as exhalation, is a crucial indicator of these sensors from the perspective of practical applications. To investigate stability, the response of the PCNFs sensor was recorded at 5 ppm and 200 ppm ethanol concentrations in a 90 RH% environment on the day of fabrication and then every 7 days over 4 weeks. As shown in Fig. 8a, the response of the PCNFs sensor exhibits minimal fluctuation and remains almost identical throughout the 4-week testing period. These changes, which are less than 1% in magnitude, indicate that the exhaled breath sensor demonstrates promising stability. Furthermore, the FESEM image depicting the surface of the PCNFs sensor (Fig. S5) subsequent to the execution of gas measurement experiments in the aforementioned environments reveals the sensor's durable stability with minimal degradation in the nanofiber structure. Furthermore, ensuring the repeatability of the PCNFs sensor's performance is another crucial factor in achieving a reliable breath sensor. The repeatability results of the PCNFs sensor in 200 ppm ethanol at 90 RH% and 6 repetitions are shown in Fig. 8b, which demonstrates the appropriate repeatability of this sensor. Therefore, the PCNFs sensor has demonstrated superior stability and repeatability, making it a promising candidate for further investigation as an exhaled breath sensor. Additionally, selectivity is one of the critical features of an exhaled breath sensor as there are many interfering gases present in the breath that could impact accurate detection^4,21,25,26^. Thus, the selectivity of the PCNFs sensor was investigated against various available gases, including ethanol, methanol, acetone, hydrogen, methane, carbon monoxide, and moisture, which are commonly found in exhaled breath and some of them are known as biomarkers for various physiological symptoms^8^. The results, shown in Fig. 8c, demonstrate that the sensor's highest response level is 90 RH%, followed by ethanol vapor with a concentration of 200 ppm. As mentioned earlier, this higher responsivity is attributed to the faster proton hopping by humidity compared to ethanol. Also, acetone has a lower response value due to its larger molecule size, lower polarity, and limited mobility, which hinders its effective participation in the proton transfer process^17,71,73^. Moreover, the sensor's surface is unable to absorb acetone efficiently, mainly due to the absence of a hydroxyl group, further reducing its response value.^17,71,73^. Other gases such as H_2_ and CH_4_, at high concentrations (> 5000 ppm) had a negligible effect on the sensor conductivity, indicating their inability to improve the electrical properties of the PCNFs sensor at room temperature^29^. Accordingly, the PCNFs sensor has the strongest selectivity to humidity and ethanol vapor, making it a promising breath sensor.

**Figure 9:**
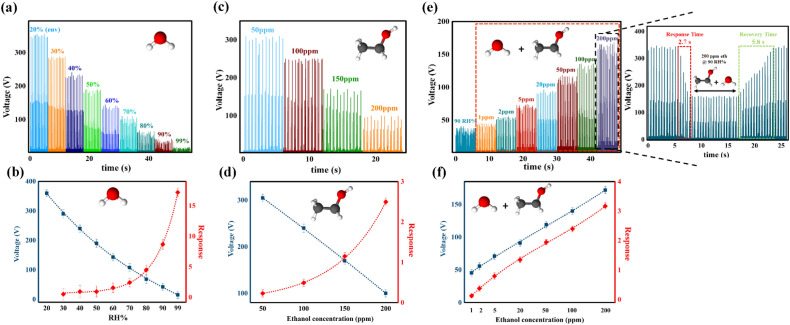
(**a**) Variation of the output voltage of the PCNFs sensor with relative humidity levels ranging from 20 to 99%; (**b**) Changes in output voltage and response values of the PCNFs sensor with relative humidity levels ranging from 20 to 99%; (**c**) Variation of the output voltage of the PCNFs sensor with ethanol concentrations of 50, 100, 150, and 200 ppm; (**d**) Changes in output voltage and response values of the PCNFs sensor with ethanol concentrations of 50, 100, 150, and 200 ppm; (**e**) Variation of the output voltage of the PCNFs sensor with ethanol concentrations of 1, 2, 5, 20, 50, 100, and 200 ppm at 90% RH (Inset: response and recovery time in 200 ppm ethanol at 90 RH%); (**f**) Changes in output voltage and response values of the PCNFs sensor with ethanol concentrations of 1, 2, 5, 20, 50, 100 and 200 ppm at 90% RH.

To achieve a self-powered exhaled breath sensor, the PCNFs-based device was integrated into parallel with a triboelectric nanogenerator to monitor the changes in circuit output voltages when the sensor was exposed to the atmospheres mentioned above (Fig. 2b). Impedance matching between the PCNFs sensor and the FTO/Kapton TENG is a critical parameter to record circuit output voltage changes upon exposure to the sensing atmospheres. As depicted in Fig. 3c, the active zone for changing the voltage of the TENG according to different resistances is in the range of 0.1–200 MΩ, which is well matched with the impedance of the PCNFs sensor. The output voltage of the PCNFs sensor at various humidity levels is presented in Fig. 9a, where the decrease in the sensor's resistance with increasing humidity results in a continuous reduction of the output voltage. Specifically, the output voltage decreases from 350 ± 5 V at a humidity level of around 20 RH% to 15 ± 2 V at 99 RH%. Similarly, as the concentration of ethanol vapor increases from 50 to 200 ppm, the output voltage decreases from 305 ± 5 to 100 ± 3 V, as shown in Fig. 9c. Also, the variation of voltage and response according to different amounts of humidity and ethanol concentration can be observed in Fig. 9b,d, respectively. Furthermore, with the increase in ethanol concentration at 90 RH%, the resistance of the sensor also increases uniformly due to the disruption of the proton hopping mechanism by ethanol vapor molecules (Fig. 9e). For this reason, by raising the ethanol concentration to 200 ppm, the output voltage enhances from 40 ± 5 V (in an atmosphere of 90 RH% without ethanol content) to 175 ± 6 V (in an atmosphere of 200 ppm ethanol at 90 RH%). This increase in output voltage and sensor response with different concentrations of ethanol is illustrated in Fig. 9f. Given that the response and recovery time are crucial performance parameters of a sensor, its values for the PCNFs sensor were assessed in an environment with 200 ppm ethanol at 90 RH%. As demonstrated in the inset of Fig. 9e, the sensor exhibited a response and recovery time of 2.7 s and 5.8 s, respectively.

**Figure 10:**
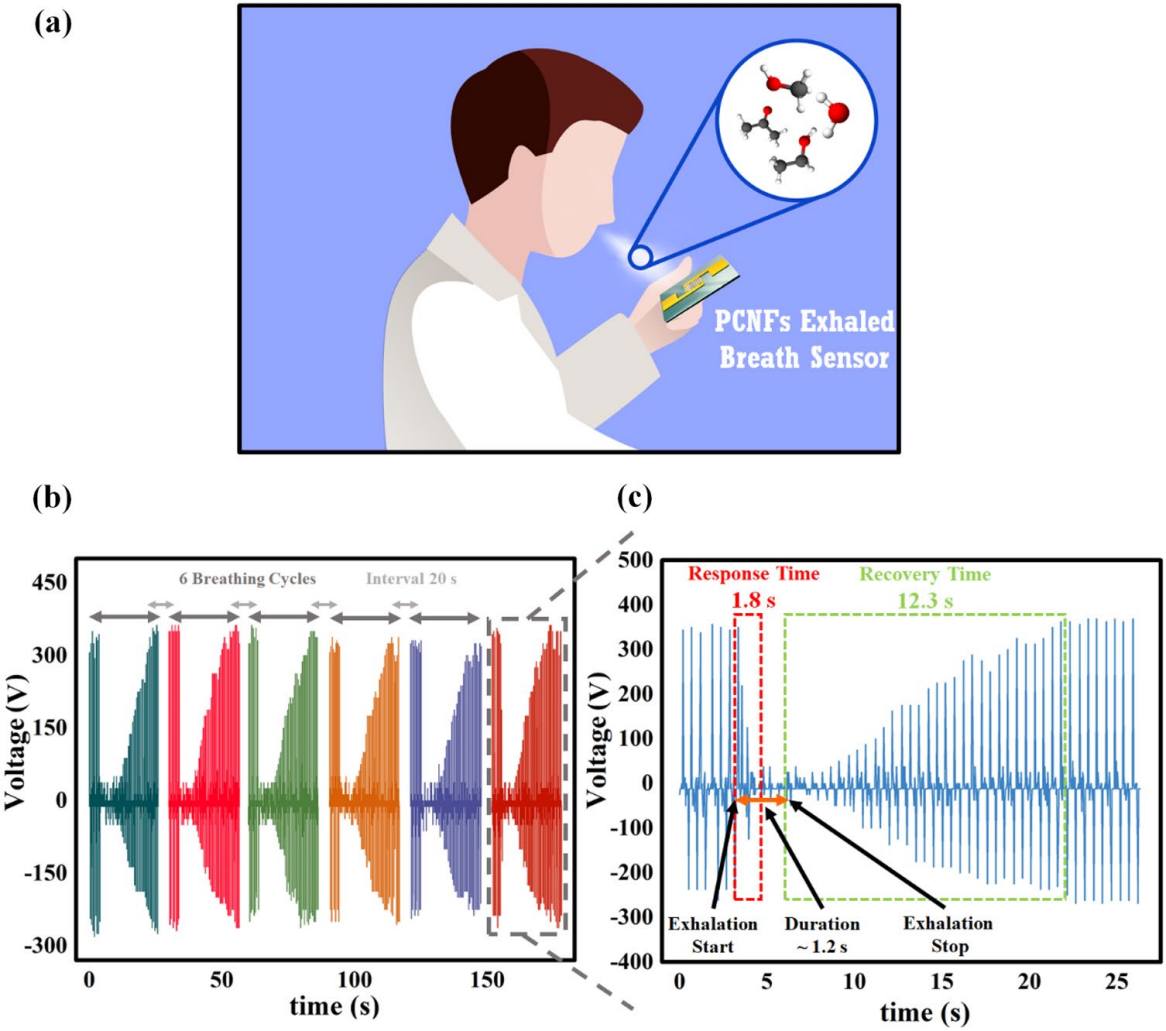
(**a**) Schematic of a natural human breath test. (**b**) The change in the output voltage of the PCNFs sensor when exposed to natural human breath; (**c**) The response and recovery time of the PCNFs sensor to natural human breath.

Finally, to demonstrate the potential of the PCNFs sensor as a self-powered exhaled breath sensor, its capability to detect humidity in natural human breath and its response and recovery times were evaluated (Fig. 10a). As the relative humidity in the atmosphere surrounding exhaled breath through the nose reaches around 90 RH%, the self-powered PCNFs sensor has the potential in respiratory monitoring through humidity detection. Figure 10b shows the decrease in TENG output voltage upon exposure to 90 RH% of exhaled breath with six repetitions, indicating appropriate repeatability of the PCNFs-based breath sensor in real-world applications. For this analysis, the sensor was placed at a distance of 10 cm from the nose, and the exhaled breath reached the sensor's surface for ~ 1.2 s with six repetitions and a 20 s interval. In addition, short response and recovery times are crucial for breath monitoring sensors in real applications. The PCNFs sensor exhibits favorable response and recovery times to exhalation, as shown in Fig. 10c, with values of 1.8 ± 0.2 s and 12.3 ± 0.2 s, respectively. This excellent performance in response and recovery time can be attributed to using composite nanofiber structures with a large surface area and the ability to adsorb and desorb gas molecules^60^. On the other hand, screening for diseases in the early stages is critical to improving survival rates and patient treatment quality^3,13,14,17^. Breath analysis has become a recognized tool for diagnosing illnesses based on changes in the concentration of specific gases in exhaled breath or the presence of biomarkers. Although the self-powered PCNFs sensor possesses vital attributes, such as high sensitivity, appropriate selectivity in the presence of interfering gases, fast response/recovery time, long-term stability, and repeatability, challenges in the breath analysis process have hindered its practical applications^4,21,25,26^. Several challenges need to be addressed before breath analysis can be used as a practical method for diagnosing illnesses. These challenges include the influence of exhalation flow rate on the sensor response, the strong dependence of exhalation gas concentration on parameters such as age, gender, weight, food habits, lifestyle, and pregnancy, changes in the concentration of multiple respiratory gases simultaneously due to disease, complicated breath sampling for meaningful analysis, and the dependence of the concentration of some gases on the breathed air of the surrounding environment and the duration of its breathing^4,9,21,26^. Despite all these challenges, breath analysis presents an innovative and less invasive method compared to traditional blood testing, which has garnered significant attention from researchers and is still in its early stages of development.

Table 1 presents a comprehensive summary of the sensing performance of the proposed PCNFs-based sensor compared to other nanomaterial-based sensors recently reported in the literature^28,29,31,46,62,63,65–67,75–79^. The results of this study demonstrate that the self-powered exhaled breath sensor based on PCNFs exhibits promising responsivity to a wide humidity range of 30–99 RH% and parts per million concentrations of ethanol in high-humidity environments. It is noteworthy that, unlike previous sensors that typically operate at temperatures exceeding 150 °C, the PCNFs-based sensor can detect ppm concentrations of ethanol at ambient temperature in 90 RH%. This is an important finding as high-temperature sensors require excessive energy consumption and can reduce equipment lifespan. Moreover, using a triboelectric nanogenerator as a power source further emphasizes the advantages of the PCNFs sensor, eliminating the need for battery replacement and making it a highly portable exhaled breath analyzer device.

**Table 1:** Comparison of sensing performance of a self-powered exhaled breath sensor based on PCNFs with other literature sensors used in comparable atmospheric conditions.

Sensing material	Sensing atmosphere	Sensing temperature (°C)	Response value	Response time/recovery time (s)	Self-powered	References
Indium-doped CuO Nanostructure	300 ppm Ethanol	116	67.1	14/20	×	^31^
Hollow Dodecahedral Cu_2_O Nanocages	100 ppm Ethanol	250	4.6	112.4/157.5	×	^65^
Hydrogenated Cu_2_O Octahededrons	100 ppm Ethanol	200	1.78	1/1.2	×	^76^
Double Shell Cu_2_O Hollow Microspheres	100 ppm Ethanol	187	6	–	×	^28^
CuO/Cu_2_O Nanolayer	100 ppm Ethanol	350	1.5	18.5/49.6		^67^
Cu_2_O Nanowires	50 ppm Ethanol	250	1.78	17/35	×	^66^
CuO/Cu_2_O/Ag Nanochannel	1 ppm Ethanol	300	5.5	–	×	^29^
Polyimide Nanowire	10,000 ppm Ethanol	Room Temp	0.85	28/60	✓	^79^
WO_3_ nanorods	100 ppm Ethanol	Room Temp	1.9	–	✓	^79^
SnS_2_/RGO	97 RH%	Room Temp	65	6/15	✓	^77^
RGO-TiO_2_	95 RH%	Room Temp	359.2	1/5.2	✓	^1^
SnS_2_ Nanosheets	90 RH%	Room Temp	4	4/7	✓	^46^
PEO/CuO/MWCNT Composite Nanofibers	90 RH%	Room Temp	43.7	3/22	×	^63^
PVA/PEO/CuO Nanocomposites	70 RH%	Room Temp	24	-	×	^62^
β-Ni(OH)_2_/MXene	100 ppm Ethanol @ 87 RH%	Room Temp	6.67	15/4	✓	^53^
CuO Nanofibers	200 ppm Ethanol	Room Temp	0.3	−	×	^75^
	200 ppm Ethanol @ 50 RH%		0.8	200/320		
PEO/Cu_2_O Composite Nanofibers	90 RH%	Room Temp	9.1	3.2/5.4	✓	This work
	200 ppm Ethanol		2.6	2.1/4.3		
	200 ppm Ethanol @ 90 RH%		3.2	2.7/5.8		

## Conclusion

In this study, a self-powered breath sensor was developed to detect ethanol levels in human exhaled breath, employing polyethylene oxide/copper (I) oxide composite nanofibers (PCNFs) and FTO/Kapton triboelectric nanogenerator as the power source. The detection of ethanol concentration in exhaled breath holds promise as a potential biomarker for the early diagnosis of lung cancer. However, the presence of interfering gases like methanol and acetone, coupled with the elevated humidity levels in exhaled breath, necessitates the creation of a sensor with high selectivity for ethanol and robust stability in high humidity conditions. Consequently, the PCNFs sensor's performance was assessed in three different atmospheres: high humidity, pure ethanol, and ethanol in the presence of 90 RH% (exhaled breath simulator). The PCNFs sensor demonstrated response values of 9.1, 2.5, and 3.2 at 90 RH%, 200 ppm ethanol in pure form, and in the presence of 90 RH%, respectively. Notably, the PCNFs-based sensor exhibited remarkable attributes such as consistent output signals, prolonged stability, exceptional selectivity, and rapid response/recovery time. Furthermore, the performance of the PCNFs-based self-powered exhalation sensor was investigated in the sensing environment of human exhaled breath to test in practical applications in terms of sensing response value, response/recovery time, and performance repeatability. Exhaled breath sensors have the potential to revolutionize the field of disease diagnosis and monitoring by providing a non-invasive and rapid diagnostic tool. The applications of exhaled breath sensors in various disease areas are still under exploration, and further research is needed to understand the potential of this technology. Nonetheless, the development of exhaled breath sensors represents a promising area of research that has the potential to improve the accuracy, speed, and convenience of disease diagnosis and monitoring.

### Supplementary Information

Supplementary Video 1.

Supplementary Video 2.

Supplementary Information 1.

## Data Availability

The datasets used and/or analyzed during the current study available from the corresponding author on reasonable request.
