# Supplementary material for: Self-powered triboelectric nanogenerator sensor for detecting humidity level and monitoring ethanol variation in a simulated exhalation environment
Source: Sci Rep. 2024 Jan 18;14:1562. doi: 10.1038/s41598-024-51862-6 (PMC10796746; doi:10.1038/s41598-024-51862-6)
Supplement: Supplementary file 3 — Supplementary Information 1. [file 41598_2024_51862_MOESM3_ESM.docx]

**Supporting information**

**Triboelectric Nanogenerator-Based Self-Powered Ethanol Sensor for Detecting Ethanol Variation in a Simulated Exhalation Environment as a Potential Biomarker of Lung Cancer**

Nima Mohamadbeigi ^a^, Leyla Shooshtari ^a^, Somayeh Fardindoost ^a, b^, Mohaddese Vafaiee ^a^, Azam Irajizad ^a, c,^ *, Raheleh Mohammadpour ^a,^ *

^a^ *Center for Nanoscience and Nanotechnology,* [*Institute for Convergence Science & Technology*](https://icst.sharif.edu/en)*, Sharif University of Technology, Tehran, Iran*

^b^ *Faculty of Engineering, Department of Mechanical Engineering, University of Victoria, P.O. Box 1700 STN CSC, Victoria, BC V8W 2Y2, Canada*

^c^ *Department of Physics, Sharif University of Technology, Azadi Street, P.O. Box 11365-9161, Tehran, Iran*


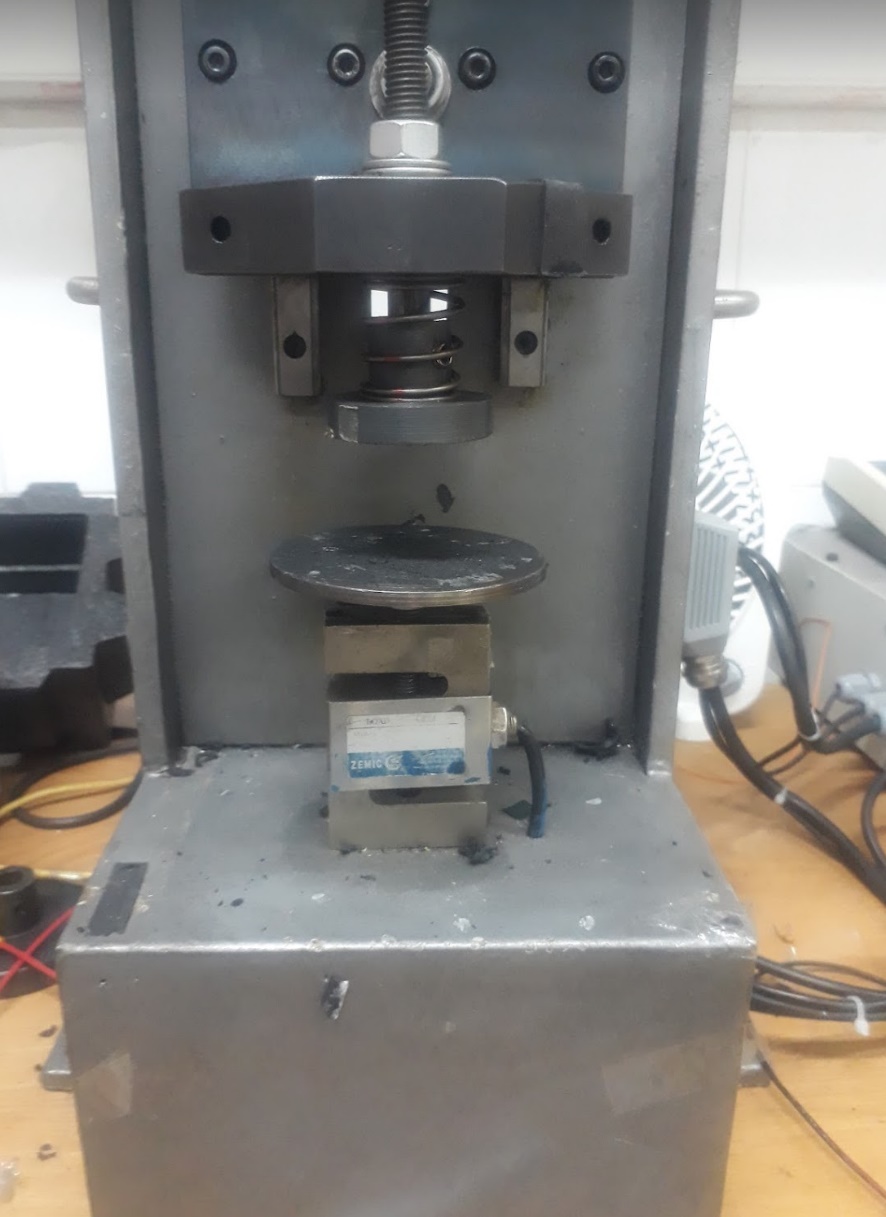


**Fig. S1:** The picture of the home-made contact separating (CS)-TENG machine

**
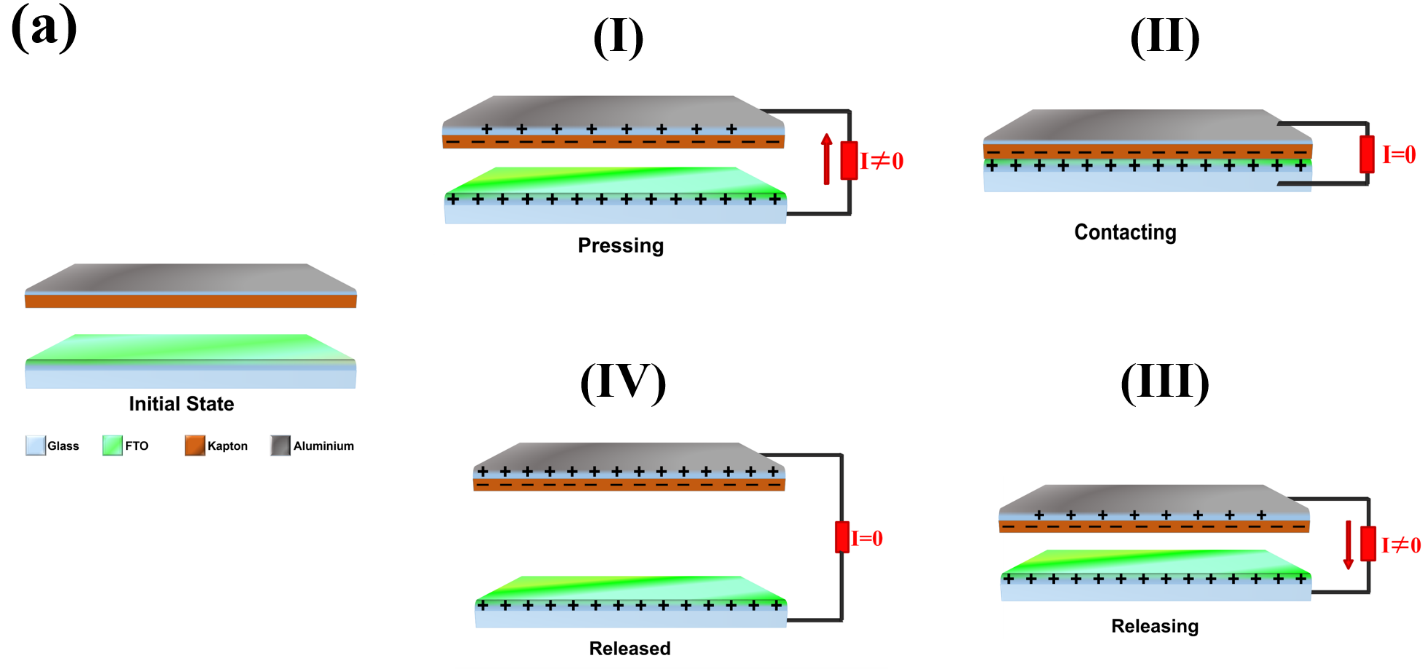
**

**
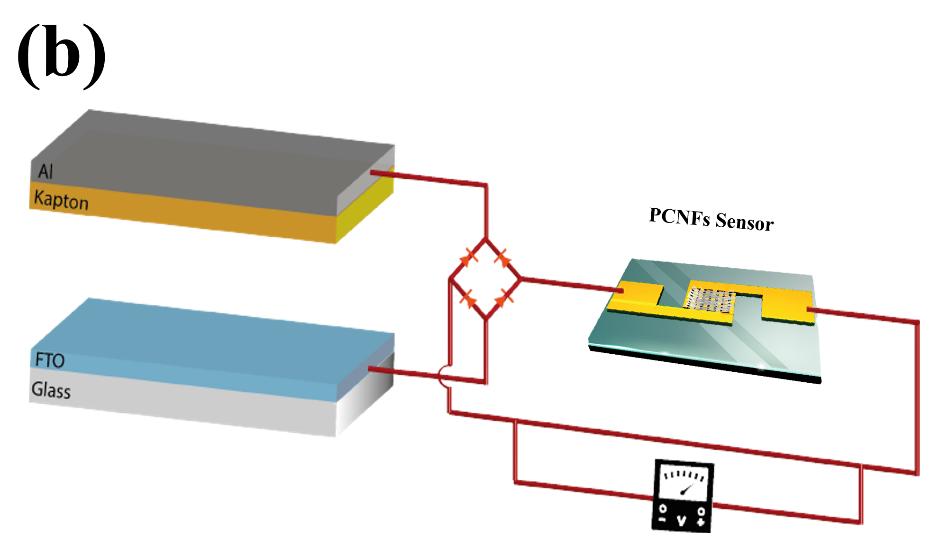
**

**Fig. S2:** (a) The principles of the electrical current generation of the FTO/Kapton TENG; (b) The employed circuit to measure the self-powered sensor voltage


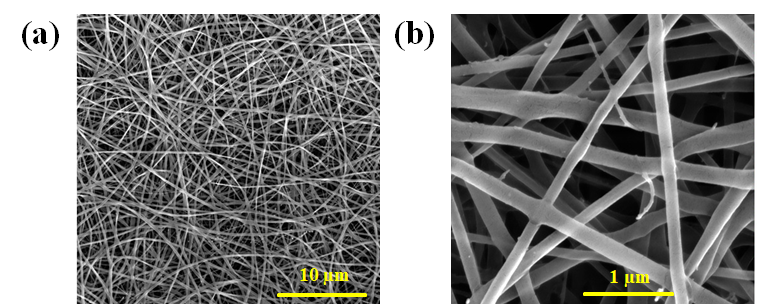


**Fig. S3:** Top view FESEM images of PCNFs at different magnifications (a) 5 kx, (b) 50 kx


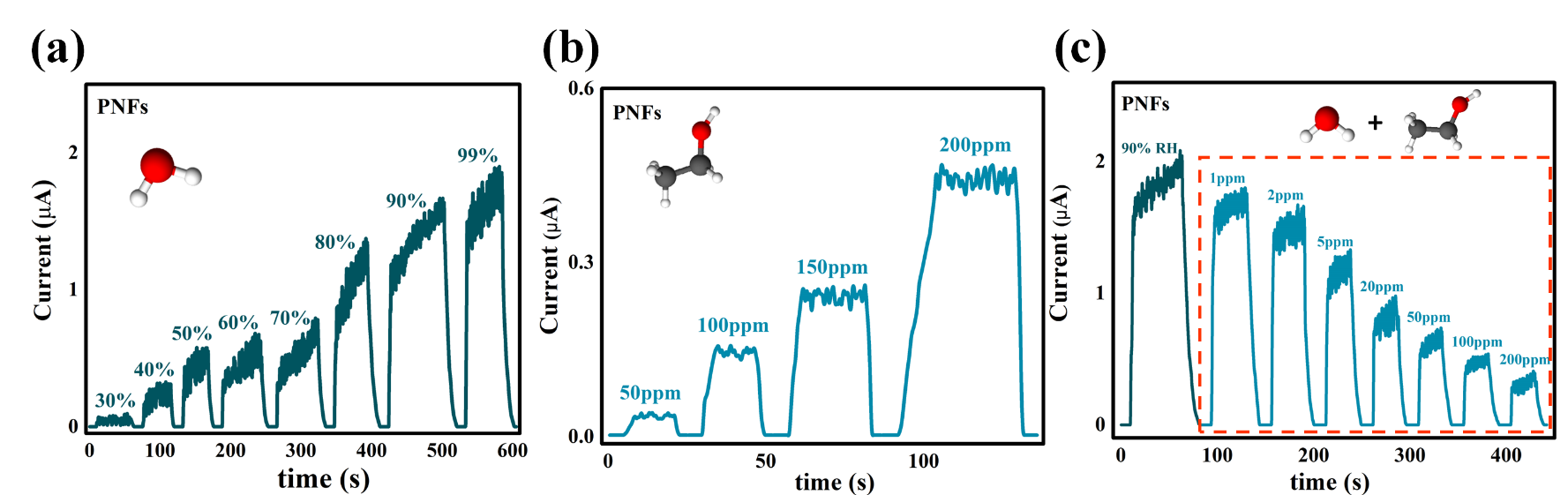


**Fig. S4:** (a) Variation in PNFs current with relative humidity ranging from 30 to 99%; (b) Variation in PNFs current with ethanol concentrations of 50,100,150 and 200 ppm; (c) Variation in PNFs current with ethanol concentrations of 1,2,5,20,50,100, and 200 ppm at 90% RH.


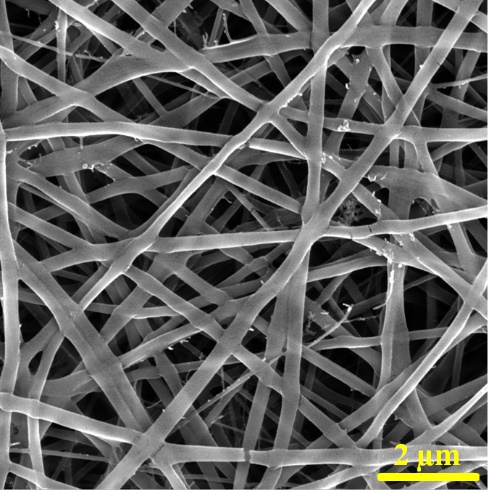


**Fig. S5:** Top view FESEM images of the PCNFs sensor after conducting the gas measurement tests at 5 ppm and 200 ppm ethanol concentrations in a 90 RH% environment, recorded every 7 days over a period of 4 weeks.
